# Tick-borne pathogens of zoonotic and veterinary importance in Nigerian cattle

**DOI:** 10.1186/s13071-016-1504-7

**Published:** 2016-04-18

**Authors:** Vincenzo Lorusso, Michiel Wijnveld, Ayodele O. Majekodunmi, Charles Dongkum, Akinyemi Fajinmi, Abraham G. Dogo, Michael Thrusfield, Albert Mugenyi, Elise Vaumourin, Augustine C. Igweh, Frans Jongejan, Susan C. Welburn, Kim Picozzi

**Affiliations:** Division of Infection and Pathway Medicine, School of Biomedical Sciences, The University of Edinburgh, Edinburgh, UK; Utrecht Centre for Tick-borne Diseases, Utrecht University, Utrecht, The Netherlands; Nigerian Institute for Trypanosomiasis Research, VOM, Jos, Plateau Nigeria; National Veterinary Research Institute, VOM, Jos, Plateau Nigeria; Royal (Dick) School of Veterinary Studies, The University of Edinburgh, Midlothian, EH25 9RG, UK; Unité d’Épidémiologie Animale, Institut National de la Recherche Agronomique, Centre de recherche de Clermont-Ferrand-Theix, France; Department of Veterinary Tropical Diseases, Faculty of Veterinary Medicine, University of Pretoria, Onderstepoort 0110, Pretoria, Republic of South Africa

**Keywords:** Cattle, Nigeria, Africa, Tick-borne diseases, Tick-borne pathogens, Zoonoses, Fulani

## Abstract

**Background:**

Ticks and tick-borne diseases undermine cattle fitness and productivity in the whole of sub-Saharan Africa, including Nigeria. In this West African country, cattle are challenged by numerous tick species, especially during the wet season. Consequently, several TBDs are known to be endemic in Nigerian cattle, including anaplasmosis, babesiosis, cowdriosis and theilerioris (by *Theileria mutans* and *Theileria velifera*). To date, all investigations on cattle TBDs in Nigeria have been based on cytological examinations and/or on serological methods. This study aimed to ascertain the occurrence of tick-borne pathogens of veterinary and zoonotic importance in cattle in Nigeria using molecular approaches.

**Methods:**

In October 2008, 704 whole blood samples were collected from indigenous cattle in the Plateau State, Nigeria. Analysis for tick-borne pathogens was conducted by means of PCR-based reverse line blotting (RLB) and sequencing targeting a panel of five genera of microorganisms (i.e. *Babesia*, *Theileria*, *Anaplasma*, *Ehrlichia* and *Rickettsia* spp.).

**Results:**

In total, 561/704 (82.6 %) animals were found infected, with 465 (69.6 %) of them being infected by two or more microorganisms, with up to 77 possible combinations of pathogens detected. *Theileria mutans* was the most prevalent microorganism (66.3 %), followed by *Theileria velifera* (52.4 %), *Theileria taurotragi* (39.5 %), *Anaplasma marginale* (39.1 %), *Anaplasma* sp. (Omatjenne) (34.7 %), *Babesia bigemina* (7.9 %), *Anaplasma centrale* (6.3 %), *Anaplasma platys* (3.9 %), *Rickettsia massiliae* (3.5 %), *Babesia bovis* (2.0 %) and *Ehrlichia ruminantium* (1.1 %). Calves were found significantly less infected than juvenile and adult cattle.

**Conclusions:**

This study provides updated, molecular-based information on cattle TBDs in Nigeria. The molecular approach employed allowed the diagnosis of numerous positive cases including carrier statuses, multiple infections and novel pathogen detections within the indigenous cattle population. Moreover, the RLB method here described enabled the detection of veterinary agents not only pertaining to bovine health, including also those of zoonotic importance.

The high prevalence recorded for *T. mutans*, *T. velifera*, *A. marginale*, *T. taurotragi* and *Anaplasma* sp. (Omatjenne), suggests they may be endemically established in Nigeria, whereas the lower prevalence recorded for other microorganisms (i.e. *A. centrale* and *B. bovis*) highlights a less stable epidemiological scenario, requiring further investigations.

**Electronic supplementary material:**

The online version of this article (doi:10.1186/s13071-016-1504-7) contains supplementary material, which is available to authorized users.

## Background

Ticks and tick-borne diseases (TBDs) threaten livestock health, welfare and productivity in the whole of sub-Saharan Africa (SSA) [1]. Nigeria is the most populous African country [2], where the cattle population is of approximately 20 million heads, eighty-per-cent of which are concentrated in the North-Central regions [3]. Here, the majority of cattle, being mostly of indigenous species (i.e. *Bos indicus*), are kept according to the traditional pastoral management of the Fulani herdsmen [4]. Reared under year-round extensive grazing, cattle are challenged by numerous tick species, especially during the wet season (i.e. June to October) when the tick burdens reach the highest abundance [5]. These ticks [i.e. *Rhipicephalus decoloratus*; *Rhipicephalus annulatus*; *Rhipicephalus guilhoni*; *Rhipicephalus geigyi*; *Hyalomma truncatum*; *Amblyomma variegatum*; *Rhipicephalus simus* Group; *Rhipicephalus turanicus*; *Rhipicephalus sanguineus* (*sensu lato*); *Hyalomma rufipes* and *Rhipicephalus lunulatus*] [6] include the vectors of pathogens of veterinary and zoonotic importance (i.e. *Anaplasma* spp., *Ehrlichia* spp., *Rickettsia* spp., *Babesia* spp. and *Theileria* spp.) [1].

Regardless of the tick burden on their livestock, the Fulani pastoralists do not usually employ acaricides, merely relying on the manual removal of the most conspicuous tick specimens from certain body sites (e.g. udder) of their cattle in order to minimise losses of milk yields due to infestation [5]. This approach, however, does not keep the animals entirely tick-free, neither does it prevent them from being re-infested nor infected by tick-transmitted pathogens [6]. Moreover, by manually removing certain tick species (i.e. *A. variegatum*), the Fulani herders are inevitably exposed to tick bites and, consequently, to the zoonotic pathogens they may transmit [e.g. spotted fever group (SFG) rickettsiae] [7–9].

Several TBDs are known to be endemic in Nigerian cattle, including anaplasmosis (by *Anaplasma marginale* mainly), babesiosis (by *Babesia bigemina* and *Babesia bovis*), cowdriosis (by *Ehrlichia ruminantium*) and theilerioris (by *Theileria mutans* and *Theileria velifera*) [10–12]. With regards to bovine anaplasmosis, the literature currently lacks confirmation of the presence of *A. centrale* in northern Nigeria [10]. In the indigenous cattle population, these TBDs are usually associated with sub-clinical or chronic conditions which are difficult to diagnose promptly in the field. However, several concomitant factors such as malnutrition, pregnancy and lactation, further concurrent infection (e.g. trypanosomiasis, haemonchosis, etc.) and/or the particularly high tick burdens of the wet season, can favour the onset of clinically apparent acute TBDs [4, 13]. Importantly, cattle can be infected by several of these pathogens simultaneously, complicating the clinical presentation and the diagnosis of TBDs [14]. Moreover, TBDs display with high morbidity and mortality in exotic cattle (i.e. *Bos taurus*) when introduced in the area for crossbreeding purposes, thus representing a major limitation to the improvement of cattle production in the country [15].

To date, all investigations of tick-borne pathogens in cattle from Nigeria have been based on cytological examinations of blood smears and lymph node biopsies [10, 11, 14–16] and/or on serological methods [15–19].

The present study aimed to investigate, by molecular means, the occurrence of tick-borne microorganisms, of both veterinary and zoonotic importance, infecting cattle in an area of North-Central Nigeria where no acaricide-based vector control is usually undertaken, and a high tick challenge and species diversity was previously documented [6]. This study relied on the application of a broad spectrum reverse line blotting (RLB) combining three different polymerase chain reaction (PCR) approaches [20–27], enabling the detection of microorganisms belonging to the genera *Babesia*, *Theileria*, *Anaplasma*, *Ehrlichia,* and *Rickettsia*.

The finding will contribute to a better understanding of the epidemiology of cattle TBDs in Nigeria, also assessing the risks for potential transmission of zoonotic pathogens to humans. Results generated would ultimately help orientate field diagnosis of bovine TBDs as well as the designing of control strategies in Nigeria, and may serve as a model for other West African countries.

## Methods

### Study area

**Fig. 1:**
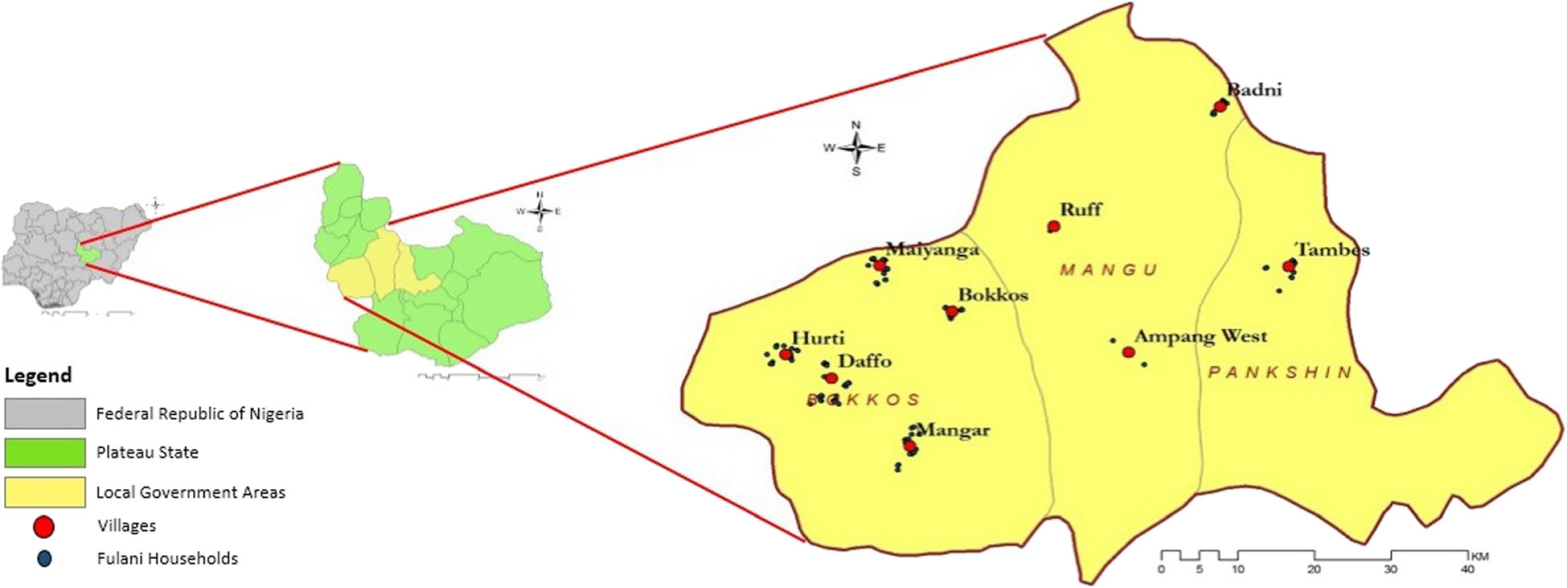
Map of the study area. Study area including the nine villages where the sampling took place. All three maps (i.e. Nigeria, Plateau State and Local Government Areas of Bokkos, Mangu and Pankshin) were designed using ArcGIS software, version 9.2

The study was conducted in nine villages belonging to three neighbouring local government areas (LGAs), namely Bokkos, Mangu and Pankshin, in the central part of Plateau State, Nigeria (9.14′–9.59′ N, 8.84′–9.38′ E), as part of a larger study focusing on trypanosomiasis [28] (Fig. 1).

The study area falls within the Northern Savannah vegetation zone, in the sub-humid region of Nigeria, where the dry season generally extends from November to April, and the wet season from April-May to October, with most (approximately 80 %) of the rains occurring between June and September [29].

All cattle reared in the area are of indigenous species (i.e. *B. indicus*), of which approximately 80 % belong to the White Fulani breed, and a smaller number are of either Bunaji or White Fulani x Rahaji crossbreeds. Cattle are grazed on communal pastures year-round according to the traditional Fulani herding system. Other livestock reared in the area include goats, sheep, pigs and poultry. In all villages, dogs are kept as household guards.

### Ethics statement

This study was carried out with the approval of each village chief, the cattle keepers, the Plateau State Ministry of Agriculture and the Ethical Committee on Animal Use and Care at the Nigerian Institute for Trypanosomiasis Research (NITR), Vom, Nigeria. All cattle enrolled in the study were selected and sampled with the agreement of their owners and the chiefs of the villages.

All sampled animals were restrained with the help of their owners and handled humanely.

### Sample collection

Seven-hundred-and-four indigenous (*B. indicus*) cattle of various sex and age (i.e. 43 calves, 184 juveniles and 477 adults) were randomly selected in nine villages, identified as a subset of a previously conducted cluster sampling [28] for being representative of the agro-ecological zone, the cattle population and management in the Plateau State, North-Central Nigeria.

Age of the animals was estimated on the basis of the dentition score method developed for zebu cattle under a low plane of nutrition [30] and on the information provided by their owners. Once quantified, each animal’s age was recorded either as ‘calf’ (0–6 months), ‘juvenile’ (6–24 months), or ‘adult’ (older than 24 months).

Whole blood samples were collected from each of the selected animals by jugular venipuncture and approximately 100 μl were applied onto Whatman FTA™ cards (Whatman Biosciences, Cambridge, UK). After being allowed to air-dry over night at room temperature, all samples were placed in foil pouches with a silica desiccant and mailed to the University of Edinburgh to be subjected to molecular processing.

### DNA extraction and elution

Once in the laboratory, a protocol for DNA extraction and dilution was followed [31]. Briefly, five three mm-circular portions of each sample-saturated matrix of each FTA™ card were punched using a Harris Micro Punch™ (Whatman BioSciences, Cambridge, UK) and placed in a sterile 1.5 ml eppendorf tube. Discs were then washed twice for 15 min in 1 ml of FTA™ purification reagent (Whatman BioSciences, Cambridge, UK) to remove any PCR inhibitors from the sample, and rinsed twice for 15 min in 1 ml of 1x Tris-EDTA buffer (Sigma-Aldrich Ltd, Dorset, UK) to remove traces of FTA™ purification reagent. Each test sample (i.e. 5 discs) was then carefully transferred to a sterile 0.2 ml PCR tube and allowed to air-dry at 37 °C for 40 min.

Afterwards, each samples’ DNA was eluted by adding 100 μl of 5 % Chelex® 100 (Sigma-Aldrich Ltd, Dorset, UK) solution to each PCR tube and incubating at 90 °C for 30 min in a Dyad Peltier thermal cycler^©^ (MJ Research Inc., USA).

### PCR

After elution, each sample was subjected to three PCR amplifications targeting a 460–540 bp long fragment from the V4 hypervariable region of the 18S ribosomal RNA (rRNA) gene for *Theileria* and *Babesia* spp. [20], a 460–520 bp long fragment from the V1 hypervariable region of the 16S SSU rRNA gene for *Ehrlichia* and *Anaplasma* spp. [21, 22], and a 350–400 bp variable region in the 16S rRNA gene for *Rickettsia* spp. [23] (see also Table 1).

**Table 1:** Primer sets employed for PCR amplification

PCR target	Primer	Sequence (5′– 3′)	Reference
*Theileria/Babesia* spp. 18S rDNA	Forward (RLB-F2)	GACACAGGGAGGTAGTGACAAG	[20]
	Reverse (RLB-R2)	Biotin-CTAAGAATTTCACCTCTGACAGT	
*Ehrlichia/Anaplasma* spp. 16S rDNA	Forward (16S8FE)	GGAATTCAGAGTTGGATC(A/C)TGG(C/T)TCAG	[21]
	Reverse (BGA1B-new)	Biotin-CGGGATCCCGAGTTTGCCGGGACTT(C/T)TTCT	[22]
*Rickettsia* spp. 16S rDNA	Forward (Rick-F1)	GAACGCTATCGGTATGCTTAACACA	[23]
	Reverse (Rick-R2)	Biotin-CATCACTCACTCGGTATTGCTGGA	

Each PCR was carried out on a total volume of 25 μl, using 5 μl of 5× Phire reaction buffer (Thermo Scientific, USA), 0.5 μl of 10 mM dNTPs (Rovalab GmbH, Germany), 0.5 μl of 20 pmol/μl of each forward and reverse primer (Integrated DNA Technologies, Inc., USA), 0.25 units of Phire Hot Start II DNA polymerase (Thermo Scientific, USA), 15.875 μl of water, and 2.5 μl of template DNA. Positive controls included 2.5 μl of DNA from *Theileria parva* (Acession No.: KJ095110), *Ehrlichia canis* (Accession No.: KJ095115), and rickettsial DNA > 98 % similar to *Rickettsia africae* (Accession No.: JX101606) [32], for the three aforementioned PCRs respectively. The 5’ of each reverse primer was labeled with a biotine ligand. Negative controls consisted of 2.5 μl of water and 5 % Chelex® 100 (Sigma-Aldrich Ltd, Dorset, UK)-eluted blank white paper. To minimize non-specific annealing, a touchdown PCR program was used. DNA amplification was carried out in a Dyad Peltier thermal cycler^©^ (MJ Research Inc., USA), with initial 30 s of DNA denaturation and polymerase activation step at 98 °C, followed by 10 cycles of 5 s denaturation at 98 °C, 5 s annealing decreasing from 67 to 57 °C at 1 °C per cycle, 7 s extension at 72 °C; 40 further cycles of 5 s denaturation at 98 °C, 5 s annealing at 57 °C and 7 s extension at 72 °C; and a final 1 min extension at 72 °C.

### Reverse line blotting (RLB)

**Fig. 2:**
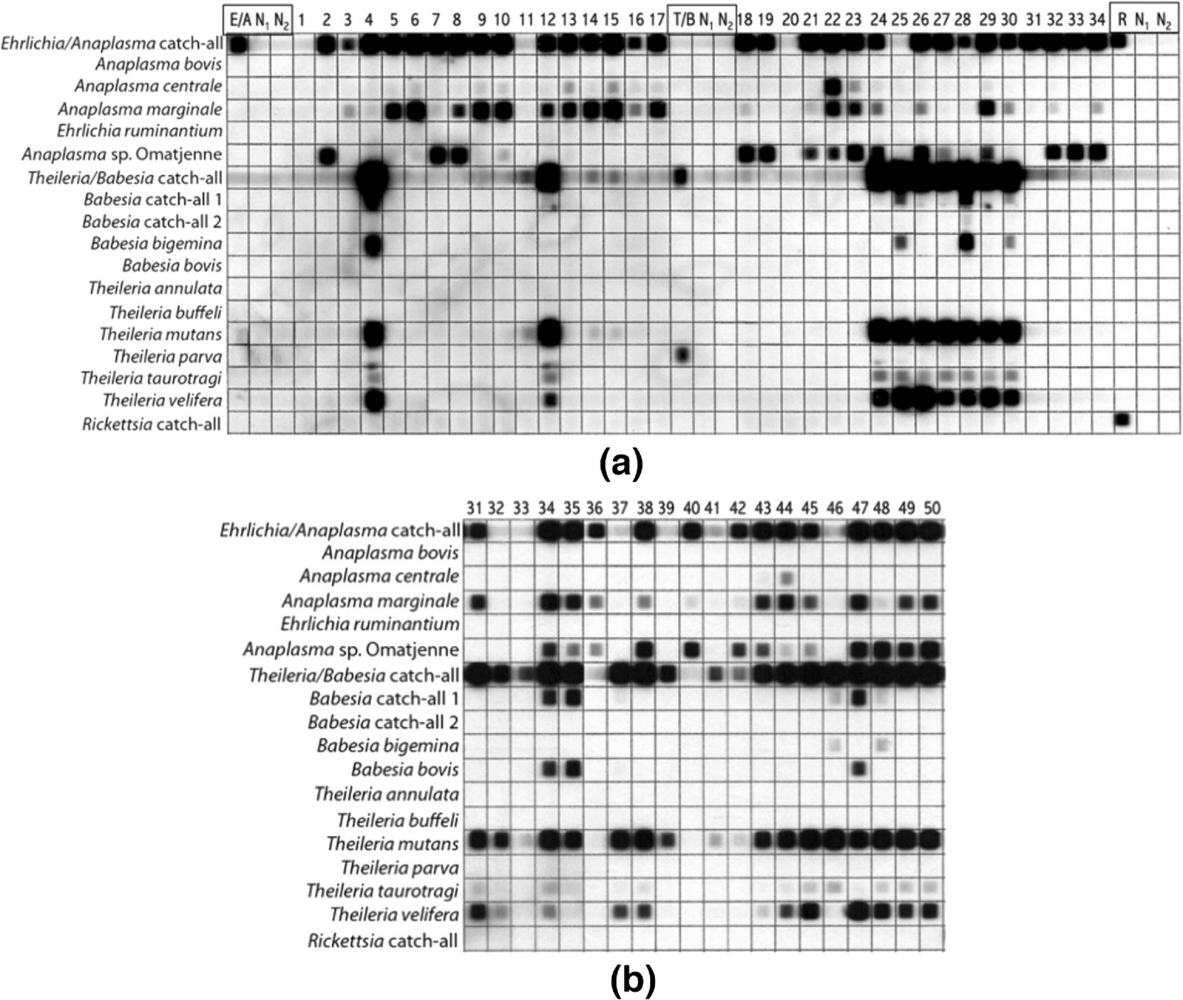
Visualization of RLB results. RLB results after X-ray development of hyperfilms, for villages of Badni (**a**): samples 1–34 and Mangar (**b**): samples 31–50. (E/A = *Ehrlichia/Anaplasma* positive control (i.e. *Ehrlichia canis*); T/B = *Theileria/Babesia* positive control (i.e. *Theileria parva*); R = *Rickettsia* positive control (i.e. *Rickettsia africae*-like); N_1_ = blank white paper negative control; N_2_ = MilliQ water control

After amplification, 10 μl of all three PCR products obtained from each individual DNA sample were mixed with 130 μl of 2xSSPE/0.1 % SDS buffer to a total volume of 160 μl. For each positive and negative controls 10 μl of their respective PCR products were diluted in 150 μl of 2xSSPE/0.1 % SDS buffer, for a total of 9 controls (i.e. 3 per each PCR) (see Fig. 2a, b).

Once prepared, samples were then heated at 100 °C for 10 min and cooled rapidly on ice. After cooling down, samples were centrifuged for 30s at 11,000 × *g* in a pre-chilled centrifuge at 4 °C. Afterwards, 160 μl of each sample and control preparation was loaded onto a Biodyne C blotting membrane (Pall Biosupport, Ann Arbor, Mich.), using a Miniblotter MN45 (Immunetics, MA, USA), on which catch-all and species-specific oligonucleotide probes (working concentration: 400 μM) containing a N-terminal *N-*(trifluoroacetamidohexyl-cyanoethyle, *N*,*N-*diisopropyl phosphoramidite [TFA])-C6 amino liker (Eurogentec, the Netherlands) were covalently linked as previously described [24].

The sequences of the nucleotide probes employed are reported in Table 2, enabling the simultaneous screening of each sample for up to five different genera and 12 species of tick-borne microorganisms. After loading them on the RLB membrane, samples were allowed to hybridize for one hour at 42 °C. Afterwards, samples were removed using aspiration and stringent washing was carried out to remove unbound PCR product as described elsewhere [24] with the modification that the first two washing steps were performed at 50 °C for 10 min to remove false annealed PCR products. Hybridized PCR products were detected by chemiluminescence reactions, using ECL reagents (Amersham, UK) after the labeling of biotin with streptavidin horseradish peroxidase. Finally reactions were visualized using ECL hyperfilm films (Amersham, UK). Development of the ECL hyperfilm was carried out with the use of an X-ray developer (Protec GmbH, Germany).

**Table 2:** Genus- and species-specific probes employed for reverse line blotting

	Tick-borne Microorganism’s Genera/Species	Probe Sequence (from 5′–3′)	Tm* (°C)	Reference
1	*Ehrlichia/Anaplasma* catch-all	GGGGGAAAGATTTATCGCTA	58	[22]
2	*Anaplasma bovis*	GTAGCTTGCTATG(A/G)GAACA	56–58	[20]
3	*Anaplasma centrale*	TCGAACGGACCATACGC	61	[22]
4	*Anaplasma marginale*	GACCGTATACGCAGCTTG	59	[22]
5	*Ehrlichia ruminantium*	AGTATCTGTTAGTGGCAG	54	[22]
6	*Anaplasma* sp. (Omatjenne)	CGGATTTTTATCATAGCTTGC	57	[22]
7	*Theileria/Babesia* catch-all	TAATGGTTAATAGGA(A/G)C(A/G)GTTG	55–59	[25]
8	*Babesia* catch-all 1	ATTAGAGTGTTTCAAGCAGAC	57	Nijhof (unpublished)
9	*Babesia* catch-all 2	ACTAGAGTGTTTCAAACAGGC	60	Nijhof (unpublished)
10	*Babesia bigemina*	CGTTTTTTCCCTTTTGTTGG	58	[24]
11	*Babesia bovis*	CAGGTTTCGCCTGTATAATTGAG	61	[24]
12	*Theileria annulata*	CCTCTGGGGTCTGTGCA	62	[20]
13	*Theileria buffeli*	GGCTTATTTCGG(A/T)TTGATTTT	56–57	[24]
14	*Theileria mutans*	CTTGCGTCTCCGAATGTT	59	[24]
15	*Theileria parva*	GGACGGAGTTCGCTTTG	60	[26]
16	*Theileria taurotragi*	TCTTGGCACGTGGCTTTT	62	[24]
17	*Theileria velifera*	CCTATTCTCCTTTACGAGT	54	[24]
18	*Rickettsia* catch-all	TTTAGAAATAAAAGCTAATACCG	54	[27]

*Tm = melting temperature

### DNA purification and sequencing

To further ascertain species identity, samples hybridizing only with a catch-all probe were subjected to DNA purification using the QIAquick Gel Extraction Kit (Qiagen GmbH) and single read sequencing via a Sanger ABI 3730xl (GATC Biotech, Germany).

Sequence inspection, cleaning and alignment were conducted manually using Bioedit (version 7.0.5.2) [33]. Sequences were then identified with the use of the Basic Local Alignment Search Tool (BLAST) (NCBI Blastn). Selected sequences amongst those obtained were deposited in GenBank on 3 December 2013.

### Statistical analysis

Prevalence of infected animals, single and multiple infections, and of each tick-borne microorganism were calculated with the R software (http://www.R-project.org) the ‘survey’ package, using the exact binomial 95 % confidence interval (CI) and after weighting according to the reciprocal of the sample size of each village. Chi-square test in the WinPepi software was used to test the null hypothesis for significant difference between age classes (i.e. calves, juveniles and adults) with regards to overall and each individual pathogen’s infection. *P*-values lower 0.05 were considered as statistically significant.

Frequency of combinations of co-infective tick-borne pathogens were calculated by normal counts according to age classes. Moreover the statistical likelihood of all possible infection patterns detectable in this study was assessed through the association screening approach [34], considering the three age classes identified (i.e. calves, juveniles and adults) altogether. Briefly, the association screening approach is a test based on a simulated theoretical distribution of a statistic and its associated confidence interval, under the null hypothesis H0 that infection patterns (i.e. parasite associations or single infections) are random. In the case of this study, the occurrence (i.e. counts) of all possible combination of parasites or single infections, was theoretically simulated, with each infection pattern (either single or type of multiple infections) being exclusive of one another. The ‘envelope ()’ function from the ‘boot’ package in the R software (http://www.R-project.org) was used to estimate the 95 % confidence envelope for the combination count distribution profile that includes all possible infection patterns. A global test based on the 95 % confidence envelope was first run. When H_0_ was rejected, the local tests based on the number of possible parasite combinations confidence intervals were performed.

For all statistical tests employed, *P*-values < 0.05 were considered as statistically significant.

## Results

### Overall infection rates

561/704 cattle (82.6 %, 95 % CI: 79.2–85.9 %) were found infected by at least one tick-borne microorganism (see Table 3).

**Table 3:** Cattle screened and found infected for any tick-borne microorganism in the study area

Village name	Total cattle population	Animals sampled (Infected)			
		Calves	Juveniles	Adults	Totals
Ampang West	790	0	33 (28)	47 (38)	80 (66)
Badni	383	4 (3)	20 (18)	56 (46)	80 (67)
Bokkos	2142	6 (6)	25 (22)	49 (42)	80 (70)
Daffo	2933	4 (1)	17 (12)	51 (45)	72 (58)
Hurti	1011	2 (1)	9 (7)	69 (64)	80 (72)
Maiyanga	2543	6 (5)	32 (31)	42 (32)	80 (68)
Mangar	1373	5 (3)	29 (25)	46 (44)	80 (72)
Ruff	154	5 (1)	18 (14)	49 (32)	72 (47)
Tambes	854	11 (6)	1 (0)	68 (35)	80 (41)
Total	12,183	43 (26)	184 (158)	477 (378)	704 (561)

### Microorganisms’ prevalence

**Fig. 3:**
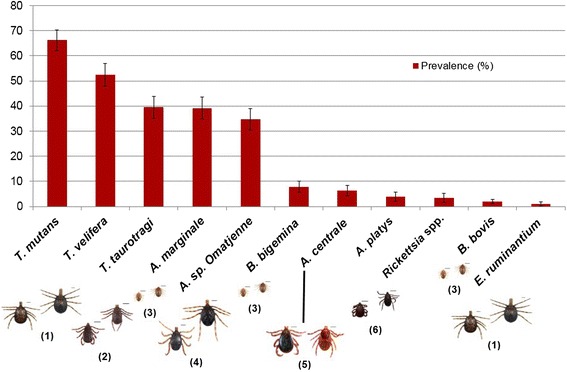
Prevalence (%) of each tick-borne pathogen in relation to its primary competent vector ticks. (1) = *A. variegatum* for *T. mutans*, *T. velifera* and *E. ruminantium*; (2) = *Rhipicephalus* spp. for *T. taurotragi*; (3) = *Rhipicephalus* (*Boophilus*) spp. for *A. marginale*, *B. bigemina*, *A. centrale* and *B. bovis*; (4) = *H. truncatum*, presumably, for *Anaplasma* sp. (Omatjenne); (5) = *Rh. simus* for *A. marginale* and *A. centrale*; (6) = *Rh. sanguineus* (*sensu lato*) for *A. platys* and *Rickettsia* spp. (*R. massiliae* according to 16S rDNA sequencing). Vector competence for the transmission of *T. taurotragi*, *Anaplasma* sp. (Omatjenne), *A. platys*, *R. massiliae*; *Anaplasma* spp. in *Rh. simus* Group and *B. bovis* in *Rh*. (*Bo*.) spp. in Nigeria needs to be further confirmed

*Theileria mutans* was the most prevalent microorganism (*n* = 435/704, 95 % CI: 62.1–70.4 %), followed by *Theileria velifera* (*n* = 348/704, 95 % CI: 47.9–56.9 %), *Theileria taurotragi* (*n* = 260/704, 95 % CI: 35.1–43.9 %), *Anaplasma marginale* (*n* = 268/704, 95 % CI: 34.8–43.5 %), *Anaplasma* sp. (Omatjenne) (*n* = 239/704, 95 % CI: 30.5–38.9 %), *Babesia bigemina* (*n* = 57/704, 95 % CI: 5.6–10.2 %), *Anaplasma centrale* (*n* = 57/704, 95 % CI: 4.2–8.3 %), *Ehrlichia*/*Anaplasma* spp. (*n* = 27/704, 95 % CI: 2.1–5.7 %), *Rickettsia* spp. (*n* = 19/704, 95 % CI: 1.7–5.2 %), *Babesia bovis* (*n* = 16/704, 95 % CI: 1.0–2.9 %), *Ehrlichia ruminantium* (*n* = 8/704, 95 % CI: 0.2–1.9 %) (see also Fig. 3).

*Theileria mutans*, *T. velifera*, *T. taurotragi*, *A. marginale* and *Anaplasma* sp. (Omatjenne) were significantly (*P* < 0.0001) more prevalent (above at least 30 %), than *A. centrale, B. bigemina, A. platys, Rickettsia* spp., *B. bovis* and *E. ruminantium* (below 10 % in prevalence).

Sequenced *Ehrlichia*/*Anaplasma* catch-all 16S rRNA positive samples were found 99-100 % similar with *Anaplasma platys* (Accession No.: KC989957.1, KF360842.1, KF576217.1) (*n* = 9). Sequenced *Rickettsia* spp. 16S rRNA gene fragments were found 100 % similar with spotted fever group (SFG) rickettsiae (i.e. *Rickettsia massiliae*, Accession No.: NR074486.1) (*n* = 3).

In all villages, the five most prevalent microorganisms were represented by *T. mutans*, *T. velifera*, *T. taurotragi*, *A. marginale* and *Anaplasma* sp. (Omatjenne). Out of the 11 microorganisms detected, only three were not found in all villages. *R. massiliae*16S rDNA was detected in seven villages (Ampang West, Bokkos, Daffo, Maiyanga, Mangar, Ruff and Tambes), *B. bovis* in four villages (Bokkos, Hurti, Mangar and Tambes) and *E. ruminantium* in only two villages (Ampang West and Bokkos).

### Co-infections

465/704 (69.6 %, 95 % CI: 65.5–73.6 %) cattle were positive for two or more microorganisms simultaneously. Overall 77 different combinations of microorganisms were found. The largest variety of co-infections was recorded in adult cattle (*n* = 58), followed by juveniles (*n* = 43) and calves (*n* = 11) (see also Additional file 1).

On the whole, the most frequent combinations included three (i.e. *T. mutans* + *T. taurotragi* + *T. velifera*) to five co-infective species (i.e. *A. marginale* + *Anaplasma* sp. (Omatjenne) + *T. mutans* + *T. taurotragi* + *T. velifera*) (Table 4 and Additional file 1). *B. bigemina* was never found infecting the same host together with *B. bovis*.

**Table 4:** Statistically significant most and least likely infection patterns of the study

Infection pattern	No. of observations	95 % Confidence interval
(A) Significantly most likely infection pattern (*P* < 0.001)		
Tm + Tt + Tv	79	10 – 44
Am + AspO + Tm + Tt + Tv	42	0 – 20
Am + AspO	33	1 – 22
AspO	30	3 – 28
Am + AspO + Tm + Tt + Tv + Bb	12	0 – 5
Ap + Tm + Tt + Tv	12	0 – 7
Am + Ac + AspO + Tm + Tt + Tv	10	0 – 5
Am + Ac	8	0–7
Am + Ac + AspO	7	0 – 6
R	6	0 – 5
(B) Significantly least likely infection patterns (*P* < 0.001)		
Am + Tm	7	11 – 46
Tm + Tt	6	8 – 44
AspO + Tm	5	6 – 38
Tv	1	10 – 47
Am + Tm + Tt	1	4 – 32
Am + Tv	0	4 – 30
AspO + Tv	0	3 – 33
Tt	0	3 – 31
Tt + Tv	0	2 – 30
Am + Tt + Tv	0	1 – 24
Am + Tt	0	1 – 23

*Abbreviations*: *Ac,* *Anaplasma centrale*; *Am,* *Anaplasma marginale*; *Ap,* *Anaplasma platys*; *AspO,* *Anaplasma* sp. (Omatjenne); *Er,* *Ehrlichia ruminantium*; *R,* *Rickettsia* spp. (*R. massiliae* according to 16S rDNA sequencing); *Bb,* *Babesia bigemina*; *Tm,* *Theileria mutans*; *Tt,* *Theileria taurotragi*; *Tv,* *Theileria velifera*

### Single infections

Ninety-six single infections were detected, amongst 561 positive cases (13 %, 95 % CI: 10.3–15.6 %), of which nine were in calves (34.6 % of positive animals), 27 in juveniles (17 % of positive animals) and 60 in adult cattle (15.8 % of positive animals). Cases of single infections were mostly represented by *T. mutans* (*n* = 32), found in 7/9 villages, *A*. sp. Omatjenne (*n* = 30) and *A. marginale* (*n* = 22), detected in all study villages, followed by *Rickettsia* spp. (*n* = 6), *A. platys* (*n* = 2), *B. bigemina* (*n* = 2), *B. bovis* (*n* = 1), and *T. velifera* (*n* = 1).

Examining statistically all infection patterns detected according to the association screening approach [34], single infections by *Anaplasma* sp. (Omatjenne) (*n* = 30/239, 12.5 % of cases of infection with *Anaplasma* sp. (Omatjenne)) and by *Rickettsia* spp. (*n* = 6/19, 31.6 % of total number of infections with *Rickettsia* spp.) were found to be significantly likely (*P* < 0.001) to occur in this study (see Table 4).

### Age-class infections

**Fig. 4:**
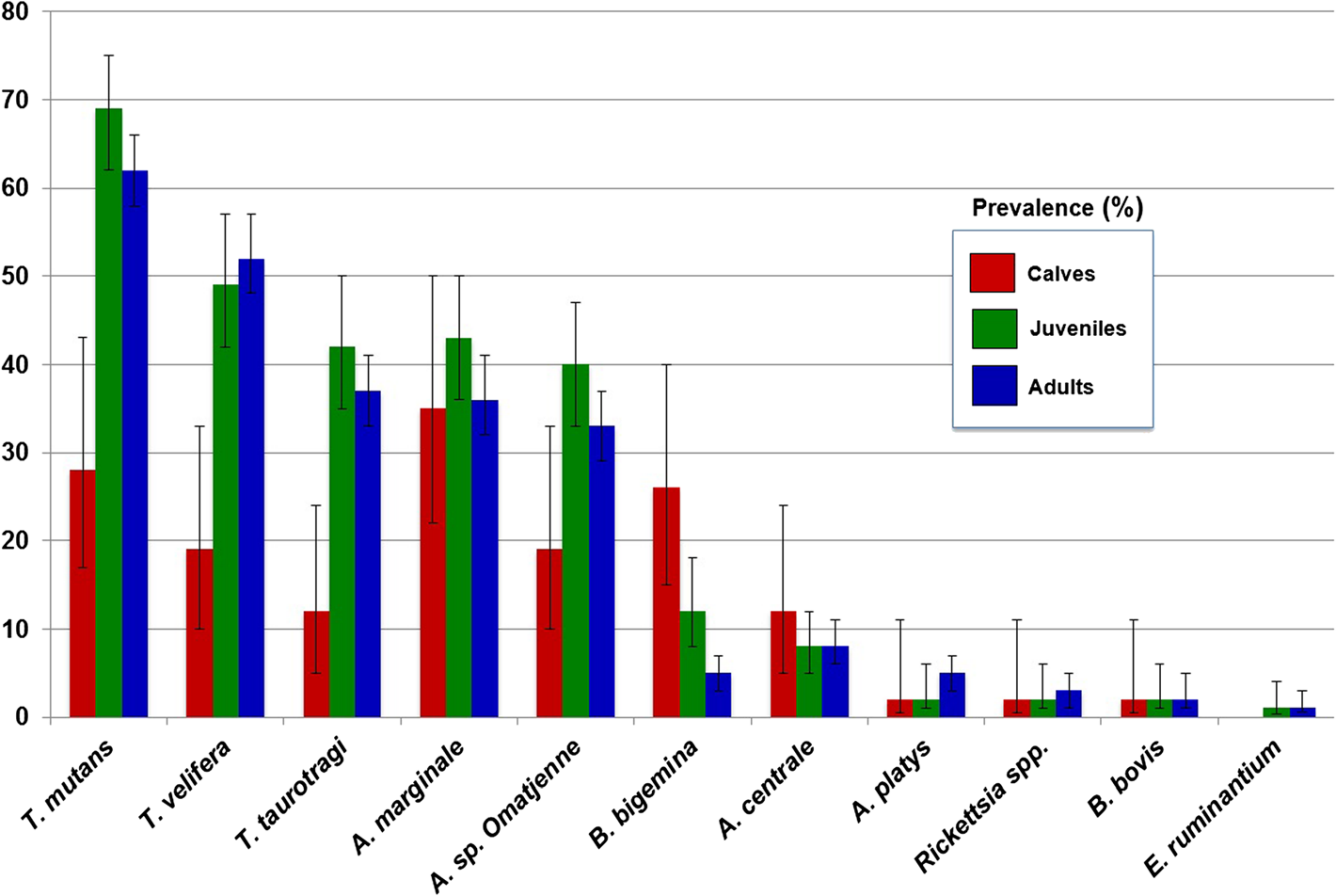
Prevalence (%) of each tick-borne pathogen detected, compared according to age classes. Error bars indicate 95 % CI, while asterisks indicate statistically significant difference between age classes. Calves were significantly less infected than both juveniles and adults for *T. mutans* (*P* < 0.0001 in both cases), *T. velifera* (*P* < 0.0001 in both cases) and *T. taurotragi* (*P* < 0.0001 and *P* = 0.001) infections. Calves were significantly less infected than juveniles (*P* = 0.02) with respect to *Anaplasma* sp. (Omatjenne) infection. Both calves and juveniles were significantly more infected than adult cattle (*P* < 0.0001 and *P* = 0.003) for *B. bigemina* infection

On the whole, calves were significantly less infected than juveniles (*χ*^2^ = 12.759, *df* = 2, OR = 0.252, *P* = 0.001) and adult cattle (*χ*^2^ = 7.096, *df* = 2, OR = 0.401, *P* = 0.02), whereas no statistically significant difference was detected between juveniles and adults (*χ*^2^ = 3.980, *df* = 2, OR = 1.592, *P* = 0.138). When reviewing each individual tick-borne infection, calves were significantly less infected than both juveniles and adults with regards to *T. mutans* (*χ*^2^ = 24.449, *df* = 2, OR = 5.756, *P* < 0.0001 and *χ*^2^ = 18.868, *df* = 2, OR = 4.225, *P* < 0.0001, respectively), *T. velifera* (*χ*^2^ = 22.728, *df* = 2, OR = 7.277, *P* < 0.0001 and *χ*^2^ = 29.616, *df* = 2, OR = 8.370, *P* < 0.0001, respectively) and *T. taurotragi* (*χ*^2^ = 16.381, *df* = 2, OR = 5.592, *P* < 0.0001 and *χ*^2^ = 13.248, *df* = 2, OR = 4.484, *P* = 0.001, respectively) infections, while no significant difference was recorded when comparing juvenile with adult cattle, for *T. mutans* (*χ*^2^ = 2.838, *df* = 2, OR = 0.734, *P* = 0.3); *T. velifera* (*χ*^2^ = 0.65, *df* = 2, OR = 1.150, *P* = 1) and *T. taurotragi* (*χ*^2^ = 1.554, *df* = 2, OR = 0.802, *P* = 0.6). In addition, calves were significantly less infected than juveniles (*χ*^2^ = 7.322, *df* = 2, OR = 2.877, *P* = 0.02), but not than adults (*χ*^2^ = 4.183, *df* = 2, OR = 2.167, *P* = 0.1), for *Anaplasma* sp. (Omatjenne). Furthermore, both calves and juveniles were significantly more infected by *B. bigemina* than adults (*χ*^2^ = 17.947, *df* = 2, OR = 0.147, *P* < 0.0001 and *χ*^2^ = 10.915, *df* = 2, OR = 0.355, *P* = 0.003, respectively). No *E. ruminantium* infection was detected in calves (see also Fig. 4).

## Discussion

The present study aimed to ascertain by molecular means the occurrence of tick-borne microorganisms of veterinary and zoonotic importance in cattle from an area of North-Central Nigeria, where no acaricide-based vector control is usually undertaken, in spite of the presence of a great species diversity and high burdens of ticks on cattle [6]. To do so, an RLB-based method enabling to test each sample against a panel of genus- (i.e. ‘catch-all’) and species-specific probes was employed (Table 2; Fig. 2a, b).

In total, 704 cattle were included in this study, the greater number of adult rather than younger cattle, among those sampled, reflects the age composition of Fulani herds, with at least 60 % of animals being adult [13, 35].

On the whole, the study disclosed high infection rates (i.e. 82.6 %) in the overall cattle population, with a broad diversity of pathogens detected, in presence of a complex scenario of frequent multiple infections. This study established the existence of a stark dichotomy in the occurrence of tick-borne pathogens, with five microorganisms, i.e. *T. mutans*, *T. velifera*, *T. taurotragi*, *A. marginale* and *Anaplasma* sp. (Omatjenne) being significantly (*P* < 0.0001) more prevalent (above at least 30 %), than each of *A. centrale, B. bigemina, A. platys, Rickettsia* spp., *B. bovis* and *E. ruminantium* (below 10 % in prevalence) (Fig. 3).

The occurrence of *Anaplasma* sp. (Omatjenne)e and *A. platys* is novel for cattle from Nigeria, and so is that of *T. taurotragi* for cattle from West Africa. Moreover, thus far, *R. massiliae* had never been found infecting livestock on a global scale.

*Theileria mutans* and *T. velifera* were the two most prevalent microorganisms (i.e. 66.3 and 52.4 %, respectively) in the study area. These two mildly pathogenic *Theileria* species have long been recognised as the only two *Theileria* species present in Nigeria [12, 15]. They are both transmitted by *Amblyomma variegatum* [36, 37] (Fig. 3), endemically present in the whole of Nigeria [5] including the Plateau State [6]. Although in different proportions, these two *Theileria* species were recorded in all age classes, being the first and second most prevalent microorganism recorded in both adults and juveniles and the second (i.e. *T. mutans*) and fourth most prevalent (*T. velifera*) microorganism detected in calves. This suggests an early exposure of cattle on the Plateau to these piroplasms, due to early infestations with *A. variegatum* [6].

*Theileria taurotragi* was the third most frequently detected microorganism in the present study (39.5 %). Primarily associated with elands [*Taurotragus* (*Tragelaphus*) *oryx* (Pallas, 1766)] in East and Southern Africa, to date it has been recorded in cattle from East, Central and Southern SSA [38]. This *Theileria* species is known to be transmitted naturally by *Rhipicephalus appendiculatus* and *Rhipicephalus zambesiensis* and, experimentally, by *Rhipicephalus pulchellus* and *Rhipicephalus evertsi* [38]. Neither the original wildlife host of this pathogen, or the aforementioned tick species, are known to be found in North-Central Nigeria, with *Rh. evertsi evertsi* being retrieved only more southward in the country [8]. Therefore, an exchange of competent multiple-host ticks from infected antelopes similar to *T. orynx* to cattle is the hypothesis here raised to explain the presence of *T. taurotragi* in cattle in Nigeria.

*Anaplasma marginale* was the fourth most prevalent pathogen detected in this study at 39.1 %. The present prevalence is comparable to that of 34 % found in the late 1970s by serological rapid card agglutination test in a similarly sized cattle population (*n* = 573) from Northern Nigeria [18]. Similarly, another serological investigation on 50 herds from ten states in Northern Nigeria disclosed a prevalence of 79.4 % of *A. marginale* [19]. These results are consistent with the present finding, considering that the use of the RLB can detect an active infection or a carrier status, but not seroconversion, detectable by means of serology.

The prevalence of *A. marginale* recorded in this study was similar in all age classes (Fig. 4). In particular, *A. marginale* was the most prevalent pathogen detected in calves, suggesting that this microorganism infects young stock early in their lives or possibly also during intra-uterinal development [39].

Persistently infecting cattle that serve as long-term reservoirs [39], *A. marginale* can be transmitted not only via the *Rhipicephalus* ticks highly prevalent in this region [6], but also via mechanical vectors, such as blood-sucking flies (*Stomoxys* spp., *Tabanus* spp.) [39], also found in this part of Nigeria [40]. Thus, calves found positive in this study seemingly represented active infections by *A. marginale*, while older cattle can be considered as chronically infected carriers.

This study provides the confirmation of the presence of *A. centrale* in North-Central Nigeria, with a prevalence of 6.3 %. Considering the richness of competent vectors (i.e. *Rh. annulatus*, *Rh. decoloratus* and *Rh. simus* Group) of this microorganism in the study area [6], one may speculate on the lower capacity of this species, compared to *A. marginale*, to disseminate within herds. Moreover, as an endemically stable setting for *Anaplasma* spp. is usually characterised by high infection rates in adult cattle, due to their carrier status, the rather homogenous prevalence detected for *A. centrale* in this study across all age classes (see also Fig. 4) may be suggestive of the existence of an extent of epidemiological ‘instability’ for this microorganism. This situation may favour the onset of sporadic episodes of acute anaplasmosis in the indigenous cattle population.

*Babesia bigemina* and *B. bovis* were documented to occur in this area, both with a prevalence below 10 (i.e. 7.9 and 2.0 %, respectively, Fig. 3). A previous serological investigation (i.e. IFAT) carried out in 50 herds from ten states in Northern Nigeria had disclosed a prevalence of 29.4 and 14.1 % for *B. bigemina* and *B. bovis* respectively [19], consistent with the present findings.

*Babesia bigemina* was known to be endemic in most of Nigeria already by the early 1920s, with cattle becoming infected in early life without showing apparent disease, then acquiring life-long immunity after recovery, due to repeated challenge by *Rh. decoloratus* ticks [10], currently the most prevalent tick species in the study area during the wet season [6]. This would explain the overall low prevalence (7.9 %) recorded in this study for *B. bigemina*, a finding that may be influenced by the lower infection rates recorded in the more numerous adults. Here, in fact, calves (25.6 %) and juveniles (12.5 %) were found significantly more infected than adult cattle (4.8 %) (*P* < 0.0001 and *P* = 0.003, respectively) (Fig. 4). It is likely that adult cattle that tested negative in this study included large proportions of animals that had successfully recovered active infections. Conversely, positive calves and juvenile cattle may be those individuals in which passive and innate immunity declined, thus developing detectable parasitaemia after exposure to sufficiently high challenge by infective *Rh. decoloratus* ticks.

The present study confirms the presence of *B. bovis* in North-Central Nigeria*.* Since the first report in 1956 in the Plateau province [10], several other studies had reported the presence of *B. bovis* in cattle in Nigeria relying on morphological [10, 11, 14] and serological (i.e. IFAT) characterization [16].

In the present study, *B. bovis* was found in a rather lower prevalence (2.0 %) than that of *B. bigemina* (7.9 %), especially in calves (*n* = 1/43 and *n* = 11/43 respectively). This could be explained considering that tick infection rates are usually higher with *B. bigemina* (0.23 %) than in *B. bovis* (0.04 %) [41], with a consequent slower rate of transmission of the latter to cattle. This would also suggest that, in an area where both species are present, endemic stability would be more likely to establish for *B. bigemina* [42]. Moreover, the lower prevalence of *B. bovis* compared to *B. bigemina* could also be explained by the seemingly lower parasitaemia levels occurring in adult carrier animals [24].

While *Rh. decoloratus* is known as vector for *B. bigemina* in Nigeria [43], the vector capacity for *B. bovis* in this country has not yet been fully clarified. A tick species that could plausibly be involved in the transmission of *B. bovis* in Nigeria is *Rh. annulatus*, known for being vector of this piroplasm in other geographic areas (e.g. Southern Europe and Northern Africa) [42], and well represented in the Plateau State [6]. *Rhipicephalus geigyi* was also found to harbour kinetes associated for shape and size with *B. bovis* [44]. If this was confirmed also for North-Central Nigeria, it would help explain the lower prevalence of *B. bovis* compared to *B. bigemina*, considering that *Rh. geigyi* is not as prevalent (7.6 %) as *Rh. decoloratus* (41.4 %) and *Rh. annulatus* (15.4 %) in the Nigerian Plateau State [6].

The very low overall prevalence (i.e. 1.1 %) detected in this study for *E. ruminantium* can be attributed to the biology of its infection. After recovery from the acute phase, low numbers of this microorganism can still reproduce in the endothelial cells of the capillaries, being released only periodically into the bloodstream [45]. The low prevalence recorded may also be related to the rigid control practice carried out traditionally by the local Fulani pastoralists, seemingly targeting specifically *A. variegatum* adults [5]. Undoubtedly, though, the manual ‘de-ticking’ of the Fulani, did not affect the detectability of *T. mutans* and *T. velifera*, also transmitted by *A. variegatum* [36, 37]. These piroplasms are nevertheless characterised by higher and longer-lasting parasitaemia in carrier animals [46] compared to *E. ruminantium* [47].

*Anaplasma* sp. (Omatjenne) was the fifth most frequently detected microorganim (34.7 %) in this study. Recently, this species was detected also in dogs sampled from this area of Nigeria [48], further confirming the circulation of this microorganism in this country.

Genetically close to *E. ruminantium*, this poorly known *Anaplasma* species was initially isolated in *Hyalomma truncatum* ticks collected from apparently healthy cattle in Namibia [49]. This tick species in known to occur in this study area [6]. Initially thought to be apathogenic in cattle, studies have also showed the association of *Anaplasma* sp. (Omatjenne) with ‘heartwater’ (cowdriosis)-like syndrome in sheep under experimental conditions [49]. Would the involvement of *A.* sp. Omatjenne in the aetiology of cowdriosis be confirmed, the low prevalence detected in the present study for *E. ruminantium* (1.1 %) would also result as more plausible, considering that this TBD was considered to be endemic in Nigeria [10, 11].

In this study, *A. platys* was found infecting cattle with a prevalence of 3.9 %; the presence of this rickettsia being documented in all the study villages. This widespread distribution of the infection suggests more an established host-pathogen relationship rather than a merely incidental finding.

*Anaplasma platys* is a thrombocytotropic bacterium usually infecting dogs, in which it is responsible for causing a syndrome known as canine infectious cyclic thrombocytopenia [50]. Recently, *A. platys* infections were molecularly detected in cattle in Sardinia, Italy [51], sheep in Senegal [52] and humans in the Americas [53, 54]; the pathogenic role of this microorganism in these hosts remains yet to be understood. The presence of this rickettsia was documented in dogs from the same region in Nigeria, with a prevalence of 6.6 % [55]. Evidence suggests that *A. platys* is vectored by *Rhipicephalus sanguineus* sensu lato [56]. This tick, whose preferential host is represented by dog [57], has also been found, though in low burdens, in cattle in this part of Nigeria [6].

The finding of 100 % similarity of *Rickettsia* spp. positive amplicons with *R. massiliae* 16S rDNA is suggestive of the occurrence of this SFG rickettsia in the study area. Future PCR and sequencing-based studies targeting SFG-specific genes (e.g. *ompA* and *ompB*) would be advisable to further confirm the occurrence of this pathogen in the Plateau State. Initially isolated in 1990 from *Rhipicephalus turanicus* and *Rh. sanguineus* (*sensu lato*) [58], *R. massiliae* is one of the most widely distributed SFG rickettsiae, described so far in all five continents [59]. The presence of this microorganism’s DNA was recently documented in questing *Rh. evertsi* ticks, collected from the vegetation in the South-western part of Nigeria [8]. In other SSA countries (i.e. Central African Republic, Guinea, Ivory Coast and Mali), *R. massiliae* was detected in several *Rhipicephalus* spp. ticks collected from cattle (e.g. *Rh. guilhoni*, *Rh. lunulatus*, *Rh. muhsamae*, *Rh. senegalensis* and *Rh. sulcatus*) [59, 60]; most of these tick species were found in this study area [6], thus representing a potential source of infection for the positive animals of the study. Would the presence of *R. massiliae* be confirmed also for the local tick fauna of Rhipicephalinae, it would raise public health concern, considering the pathogenicity of this species to humans [59].

On the whole, calves were significantly less infected than both juvenile (*p* = 0.001) and adult cattle (*P* = 0.02). This is in line with the concept of ‘inverse age resistance’, consisting in the higher resistance or tolerance to an infection of young cattle compared to adults in an endemic area for a certain TBD [61]. This is consistent with several other studies carried out in SSA, including North-Central Nigeria, where a lower proportion of infection (14.5 %) was detected in younger cattle compared to adults (36.3 %), although an index of quantification of age is not given [14].

The finding in the present study suggests that cattle are more likely challenged by the infection between six months and two years, as a possible result of the declining of a previous colostral and perhaps also innate immunity [47]. Conversely, the rather similar prevalence recorded in juvenile and adult cattle (39.7 and 33.1 %, respectively), suggests the persistence of a carrier status in older animals. Moreover, the lower infection rates in calves can be attributed to their lower tick burdens compared to juvenile and adult cattle [6].

The reported combinations of co-infective agents are suggestive of the absence of competition, or antagonist effect, among the three *Theileria* species altogether (i.e. *T. mutans*, *T. velifera* and *T. taurotragi*); these and *A. marginale* and *A*. sp. Omatjenne; between the latter two species; among the five aforementioned microorganisms, with or without *B. bigemina* or *A. centrale* (Table 4 and Additional file 1).

*Anaplasma marginale* and *Anaplasma* sp. (Omatjenne), the fourth and fifth most frequently detected microorganisms, were found to be likely (*P* < 0.001) associated with the three theilerias (i.e. *T. mutans*, *T. velifera* and *T. taurotragi*), only if present together. This may suggest a synergism between these two co-infection patterns (i.e. *A. marginale* + *Anaplasma* sp. (Omatjenne) and *T. mutans* + *T. taurotragi + T. velifera*). The frequent association between *T. mutans* and *T. velifera*, may be related to transmissions through the same tick vector (i.e. *A. variegatum*) and to the fact that infections by both are characterized by durable carrier statuses [36, 37].

*Theileria taurotragi* was always detected in presence of other *Theileria* species (Additional file 1). Indeed, while the combination of all three theilerias (i.e. *T. mutans* + *T. velifera* + *T. taurotragi*) together was found to be a statistically significant type of association (*P* < 0.001) (Table 4), the association of *T. taurotragi* with *T. mutans* (recorded in six samples only) as well as that of *T. taurotragi* with *T. velifera* (never detected in this study) were found to be statistically unlikely (*P* < 0.001) to occur in this study (Table 4). This suggests a possible favouring role played by *T. mutans* and *T. velifera*, together, towards the establishment of a parasitaemia by *T. taurotragi*. Some of these findings are consistent with those of another RLB-based study carried out in indigenous cattle from Kenya [62]; however, the frequent and statistically significant association observed between *A. marginale* and *Anaplasma* sp. (Omatjenne) was in contrast to this previous work.

In accordance with another RLB-based study in 477 cattle in Mozambique [63], no case of co-existence of the two *Babesia* spp. was recorded in this study; the lack of co-infections detected could be the hypothetical existence of competition between *B. bigemina* and *B. bovis*.

In this study, single infections accounted for less than 1/5 of the total number of positive cases. Single infections were frequent in calves (34.6 % of positive animals), followed by juveniles (17 %) and adult cattle (15.8 %), a finding that is in accordance with the principle of inverse age resistance aforementioned.

The large majority (*n* = 84/92, 91 %) of the single infections detected in this study were caused by *T. mutans*, *Anaplasma* sp. (Omatjenne) and *A. marginale*, reflecting the high prevalence of these microorganisms; and by *R. massiliae* (*n* = 6/19, 31.6 % of total number of *Rickettsia* spp. positive cases). Considering its overall low prevalence (i.e. 3.5 %) in this study, it is possible that the detectability of *Rickettsia* spp. in the blood stream may be favoured by the absence of other tick-borne pathogens.

## Conclusions

In conclusion, this study discloses the occurrence of numerous tick-borne pathogens of veterinary and zoonotic importance in cattle from Nigeria, in the presence of a complex scenario of multiple infections. The high prevalence and the great variety of pathogens recorded (including, amongst others, *T. mutans*, *T. velifera*, *A.marginale* and *B. bigemina*), poses a serious threat to the possible introduction of exotic taurine (i.e. *B. taurus*) breeds in the area.

The RLB technique employed proved to be a very sensitive tool, enabling the simultaneous detection of several microorganisms as well as the identification of pathogens not expected in this geographic area (i.e. *T. taurotragi*) and in the cattle host (i.e. *A. platys* and *R. massiliae*, based on 16S rDNA detection). Future research endeavors may incorporate species-specific probes targeting the latter two microorganisms to allow their prompt identification via RLB.

Herein, results highlight the need to consider co-infections, as opposed to single pathogens, in rural settings of extensive grazing. In particular, these findings point out the involvement of cattle in the epidemiology of tick-borne infections pertaining to dog (i.e. *A. platys*) and potentially also human health (i.e. *A. platys* and *R. massiliae*).

Future studies aiming to better understand the vectors linked to the host-microorganisms associations disclosed (i.e. *T. taurotragi*, *Anaplasma* sp. (Omatjenne), *A. platys* and *R. massiliae*) or confirmed (i.e. *B. bovis*) are also recommended. In particular, further molecular work should be warranted to confirm the occurrence of *R. massiliae* in this area, to better understand the risk of exposure for the local population handling cattle (e.g. pastoralists, veterinary and para-veterinary personnel), thus more vulnerable to tick bites.

## Additional file

Additional file 1: Multiple infections by tick-borne pathogens according to age classes and overall number of animals. (PDF 19 kb)
