# Supplementary material for: Tick-borne pathogens of zoonotic and veterinary importance in Nigerian cattle
Source: Parasit Vectors. 2016 Apr 18;9:217. doi: 10.1186/s13071-016-1504-7 (PMC4836144; doi:10.1186/s13071-016-1504-7)
Supplement: Additional file 1: — Multiple infections by tick-borne pathogens according to age classes and overall number of animals. (PDF 19 kb) [file 13071_2016_1504_MOESM1_ESM.pdf]

Supplementary Table. Multiple infections by tick-borne pathogens according to age classes and overall number of animals.

Ac = *Anaplasma centrale*; *Am* = *Anaplasma marginale*; *Ap* = *Anaplasma platys*; AspO = *Anaplasma* sp. Omatjenne; *Er* = *Ehrlichia ruminantium*; *R* = *Rickettsia* spp. (*Rickettsia massiliae* according to 16S rDNA sequencing); *Bb* = *Babesia bigemina*; *Bbov* = *Babesia bovis*; *Tm* = *Theileria mutans*; *Tt* = *Theileria taurotragi*; *Tv* = *Theileria velifera*.

	**Tick-borne pathogen species combinations**	**Number of detections**			**Totals**
		**Calves**	**Juveniles**	**Adults**	
**1**	*Tm_Tt_Tv*	1	13	65	79
**2**	*Am*_AspO_*Tm_Tt_Tv*	1	14	27	42
**3**	*Tm_Tv*	-	8	26	34
**4**	*Am*_AspO	-	4	29	33
**5**	*Am_Tm_Tt_Tv*	-	9	18	27
**6**	*Am*_AspO_*Tm_Tv*	-	6	15	21
**7**	*Ap_Tm_Tt_Tv*	-	2	10	12
**8**	*Am*_AspO_*Tm_Tt_Tv_Bb*	-	5	7	12
**9**	*Am_Tm_Tv*	-	2	9	11
**10**	*Am*_AspO_*Tm*	-	7	3	10
**11**	*Am_Ac*_AspO_*Tm_Tt_Tv*	-	1	9	10
**12**	AspO_*Tm_Tt_Tv*	-	-	10	10
**13**	*Am_Ac*	1	1	6	8
**14**	AspO_*Tm_Tv*	-	1	7	8
**15**	*Tm_Tt_Tv_Bb*	-	4	4	8
**16**	*Ap_Tm_Tv*	-	-	7	7
**17**	*Am_Tm*	1	1	5	7
**18**	*Am_Ac*_AspO	-	2	5	7
**19**	*Am_Ac_Tm_Tt_Tv*	-	3	3	6
**20**	*Tm_Tt*	-	5	1	6
**21**	*Eo_Tm*	-	4	1	5
**22**	*Eo_Tm_Tv_Bb*	-	5	-	5
**23**	*Am_Ac_Tm*	1	2	1	4
**24**	*Am_Tm_Tt_Tv_Bb*	1	3	-	4
**25**	*Am_Ac_Tm_Tv*	-	-	3	3
**26**	*Am*_AspO_*Tm_Tt_Tv_Bbov*	-	1	2	3
**27**	AspO_*Bb*	-	2	1	3
**28**	AspO_*Tm_Tt*	-	2	1	3
**29**	AspO_*Tm_Tt_Tv_Bb*	-	-	3	3
**30**	AspO_*Tm_Tt_Tv_Bbov*	-	1	2	3
**31**	*R_Tm_Tv*	-	2	1	3
**32**	*Tm_Tt_Bb*	-	2	1	3

**33**	*Tm_Tv_Bb*	1	1	1	3
**34**	*Ap_Tm*	-	1	1	2
**35**	*Ap_Tm_Tt*	-	-	2	2
**36**	*Ap_Tm_Tt_Tv_Bb*	-	-	2	2
**37**	*Ac_*AspO	-	-	2	2
**38**	*Am_Bb*	2	-	-	2
**39**	*Am_Ac_*AspO*_Tm*	-	-	2	2
**40**	*Am_Ac_Er_Tm_Tt_Tv*	-	-	2	2
**41**	*Am_Tm_Tt_Tv_Bbov*	-	1	1	2
**42**	*Am_Eo_Tm_Bbov*	1	-	1	2
**43**	*Am_Ac_*AspO*_Tm_Tv*	-	-	2	2
**44**	*Am_*AspO*_Tm_Tt*	-	2	-	2
**45**	*Am_*AspO*_Tm_Tt_Bb*	-	1	1	2
**46**	*Am_Ac_Er_*AspO*_Tm_Tv*	-	1	1	2
**47**	*Am_R_Tm*	-	2	-	2
**48**	*Ac_Tm_Tt_Tv*	-	2	-	2
**49**	AspO*_R_Tm_Tv*	-	-	2	2
**50**	*Tm_Bb*	1	-	1	2
**51**	*Tm_Tt_Tv_Bbov*	-	-	2	2
**52**	*Ap_Tm_Tt_Bb*	-	1	-	1
**53**	*Ap_Tm_Tv_Bb*	-	-	1	1
**54**	*Am_Ac_*AspO*_Tv*	1	-	-	1
**55**	*Am_Ac_*AspO*_Bb*	1	-	-	1
**56**	*Am_Ac_Er_*AspO	-	1	-	1
**57**	*Am_Ac_Tm_Bb*	1	-	-	1
**58**	*Am_Tm_Tt*	-	-	1	1
**59**	*Am_Tm_Tt_Bb*	1	-	-	1
**60**	*Am_Tm_Tv_Bb*	-	-	1	1
**61**	*Am_*AspO*_Tv*	-	1	-	1
**62**	*Am_Ac_*AspO*_Tm_Tt_Tv_Bbov*		-	1	1
**63**	*Am_Ac_*AspO*_R_Tm*	-	-	1	1
**64**	*Am_*AspO*_TM_TV_Bbov*	-	1	-	1
**65**	*Am_Ac_*AspO*_Tm_Tv_Bb*	-	1	-	1
**66**	*Am_*AspO*_R_Tm_Tv*	-	-	1	1
**67**	*Am_R_Tm_Tv*	-	-	1	1
**68**	*Am_R_Tm_Tt_Tv*	-	-	1	1
**69**	*Ac_Tm_Tt_Tv_Bb*	1	-	-	1
**70**	AspO*_R*	-	-	1	1
**71**	AspO*_Tv_Bb*	1	-	-	1
**72**	AspO*_Tm_Tt_Bb*	-	1	-	1
**73**	*Er_Tm*	-	-	1	1
**74**	*Er_Tm_Tt_Tv*	-	-	1	1
**75**	*Er_Tm_Tv*	-	-	1	1
**76**	*R_Tm_Tt_Tv*	-	-	1	1
**77**	*Tv_Bb*	-	1	-	1
